# Family APGAR and treatment outcomes among HIV patients at two ART Centres in Kumasi, Ghana

**DOI:** 10.4314/gmj.v56i3.5

**Published:** 2022-09

**Authors:** Nana K Ayisi-Boateng, Anthony Enimil, Akye Essuman, Henry Lawson, Aliyu Mohammed, Douglas O Aninng, Emmanuel A Fordjour, Kathryn Spangenberg

**Affiliations:** 1 Department of Medicine, School of Medicine and Dentistry, Kwame Nkrumah University of Science and Technology, Kumasi, Ghana; 2 University Hospital, Kwame Nkrumah University of Science and Technology, Kumasi, Ghana; 3 Department of Child Health, School of Medicine and Dentistry, Kwame Nkrumah University of Science and Technology, Kumasi, Ghana; 4 Family Medicine Unit, Department of Community Health, University of Ghana Medical School, Accra, Ghana; 5 Department of Epidemiology and Biostatistics, School of Public Health, Kwame Nkrumah University of Science and Technology, Kumasi, Ghana; 6 Department of Modern Languages, Kwame Nkrumah University of Science and Technology, Kumasi, Ghana; 7 Family Medicine Directorate, Komfo Anokye Teaching Hospital, Kumasi, Ghana

**Keywords:** Anti-Retroviral Therapy, Family APGAR, Family Functionality, HIV, Viral Suppression

## Abstract

**Objectives:**

This study aimed to examine the association between Family Adaptability, Partnership, Growth, Affection and Resolve (Family APGAR) and HIV treatment outcomes.

**Design:**

A cross-sectional study using the Family APGAR questionnaire

**Setting:**

The study was conducted in Kumasi, Ghana, at the Komfo Anokye Teaching Hospital and the Kwame Nkrumah University of Science and Technology Hospital

**Participants:**

Consenting HIV-positive patients who had been on treatment for at least 12 months were recruited.

**Main outcome measures:**

The Family APGAR questionnaire was administered, and relevant data were extracted from hospital records and analysed using STATA^®^ software. The relationship between Family APGAR and treatment outcomes was determined using Chi-squared tests or Fisher's exact test.

**Results:**

Approximately 70.1% of 304 participants were females with a mean age of 41.8 years (±9.9). At treatment initiation, 47.4% of the patients presented at World Health Organisation (WHO) clinical stages I and II and had a CD4 count ≥ 200 cells/mm^3^. Females were less likely (Odds Ratio= 0.52; 95% CI=0.31 – 0.90, p = 0.018) to report late for treatment compared with the males. After 12 months of treatment, approximately 70% recorded undetectable viral load. Patients with functional families constituted 70.4%, which had a statistically significant relationship with viral load (p = 0.041).

**Conclusion:**

HIV care providers should incorporate family functionality evaluation into clinical practice and provide early essential support to enhance treatment outcomes.

**Funding:**

None declared

## Introduction

By 2020, the 90-90-90 agenda to end the Human Immunodeficiency Virus/Acquired Immunodeficiency Virus pandemic was targeted by the Joint United Nations Programme on HIV/AIDS (UNAIDS).^1^ Despite global efforts, HIV incidence is increasing in 101 countries, 74 developing nations.^2^ In 2019, out of 38 million people living with HIV globally, only 81% were diagnosed, 67% received antiretroviral therapy (ART), and 59% had achieved viral suppression.^3^

There is a growing need to consider other factors influencing HIV/AIDS outcomes apart from drug treatment and adherence.

The primary objective in initiating highly active antiretroviral therapy (HAART) is to achieve viral suppression and undetectable HIV Ribonucleic Acid (RNA) viral load.^4^ HIV RNA levels persistently below 20 to 75 copies/ml are termed optimal viral suppression.^5^

Within 8 to 24 weeks, this is expected to be achieved by patients who adhere to antiretrovirals (ARVs), provided they do not harbour any resistant strains of HIV.^5^

The World Health Organisation (WHO) defines treatment failure as persistently detectable viral load exceeding 1000 copies/ml after at least six months of using ART^8^ and recommends viral load testing as the most preferred method for diagnosing treatment failure.^6^ However, facilities for viral load testing are lacking in many developing countries.^7^,^8^

Every individual belongs to a family and derives support from the family during ill health, incapacitation or hospitalisation.^9^ This is more pertinent within the African family setting, where the family is also expected to seek and provide for the welfare of its members. However, an HIV-infected person stands the risk of family rejection and may be stigmatised or accused of promiscuity.^9^ The family remains the bedrock of every social system^9^ and has been cited as a protective factor against stigma and discrimination.^10^ Patients who experience family dysfunction, poor family cohesion and little or no family support have poor disease control and often delay accessing health care.^11^ The family APGAR is a tool used to assess family function in five main domains; Adaptability, Partnership, Growth, Affection and Resolve.^12^

Commonly, HIV research has focused on prevalence,^5^,^13^ disclosure,^14^ couple testing,^15^ transmission prevention^16^ and medication adherence.^17^ However, there is a paucity of data on studies incorporating family dynamics into the care of chronically ill patients. Determining the effect of an illness on a patient's entire family will enable healthcare providers to address the full impact of chronic diseases,^18^ including HIV/AIDS. This study was conducted to assess family functionality's influence on virologic and CD4 count response among HIV patients.

## Methods

### Study setting and population

The study was conducted at the HIV Clinic, Komfo Anokye Teaching Hospital (KATH) and the Infectious Disease Unit (IDU) of the University Hospital, Kwame Nkrumah University of Science and Technology (KNUST), Kumasi, Ghana. Both institutions have a patient population with varied socio-demographic characteristics. Though located in Kumasi, a cosmopolitan city, both study sites receive referrals from towns and villages beyond Ashanti Region. KATH is a public institution owned by the Ministry of Health and provides tertiary-level care as a teaching hospital. Its HIV clinic, the first in Kumasi, was set up in 2003 and attends to an average of 1000 patients monthly. The IDU, established in the year 2010, is located on the campus of KNUST and provides services for students, staff, and their dependents, as well as patients who are not members of the University community. An average of 240 patients a month are seen at the IDU. Study participants were people living with HIV (PLWH) who were 18 years old and above, registered at either of the two study sites and had been on ART for at least 12 months. Participants could understand the study protocol and provided informed consent. Those excluded were HIV-positive patients on hospital admission or those who were too ill to participate.

### Study design and sampling

This was a cross-sectional study involving PLWH who visited the health facilities and collected their data at treatment initiation and 12 months after treatment. The study period was three months. Study participants were patients who reported to the study sites on clinic days for a refill of medications or to be seen by a doctor. A systematic random sampling method was used. Selected patients were screened to ensure the eligibility criteria were met. Data on clinical parameters such as HIV serotypes, WHO clinical stage, co-infections, CD4 count, and viral load were extracted from participants' hospital folders. CD4 count and viral load results of patients at both KNUST and KATH were derived from the serology laboratory at KATH. Data on participants' socio-demographic characteristics and family functionality were obtained by administering a structured questionnaire which included the Family APGAR. Hence, family functionality was assessed in participants who had been on treatment for at least 12 months. The Family APGAR is a validated tool which assesses five domains of family function: a respondent's satisfaction with family support in troubling times (Adaptability); satisfaction with family communication and problem sharing (Partnership); satisfaction with family support when pursuing new activities (Growth); satisfaction with family's expression of affection and satisfaction with family time spent together (Resolve). There are three types of responses which are graded as ‘almost always’ (2), ‘some of the time’ (1) and ‘hardly ever’ (0) It has a total score of 10 and each respondent is categorised into a severely dysfunctional family (score 0 – 3), a moderately dysfunctional family (score of 4 - 6) and a highly functional family (score of 7 – 10).^12^ The questionnaire was translated into Twi (a local language) and back-translated into English to ensure effective communication with participants who could not understand English. The translated questionnaire was assessed for construct validity by linguistic experts in the Department of Modern Languages, Kwame Nkrumah University of Science and Technology. The questionnaire was interviewer-administered. Secondary data was collected using a data capturing sheet, entered into Microsoft Excel and exported to STATA version 14 for analysis.

### Sample size estimation

An estimated 76.8% of adults living with HIV had a baseline CD4 count of less than 200 cells/mm^3^,^19^ considering a 95% confidence interval, a 5% allowable margin of error, and a non-response rate of 10%, 304 participants were recruited into the study.

### Estimation of Viral Load

This study estimated viral load in 1.1ml of plasma based on a procedure recommended by the reagents manufacturers (COBAS AmpliPrep/COBAS Taqman HIV-1 Quantitative Test, v2.0, Roche Diagnostic GmbH, Germany).

### Definition of Viral Suppression

Those who recorded viral load ≤ 1000 copies/ml were classified as viral suppression, and viral load > 1000 copies/ml was considered a virologic failure. Those who recorded viral load < 20 copies/ml were considered undetectable levels based on WHO recommendation.^21^

### Statistical Analysis

Means, standard deviations, frequencies, and percentages were used to describe the data. Chi-square or Fisher's exact tests were used to examine factors associated with the treatment outcome variables (viral load and CD4 count). Binary logistic regression was used to assess the magnitude of association of factors that showed some level of association (minimum of p<0.05) with the treatment outcomes. Crude Odds ratios (OR) and adjusted odds ratio (aOR) at 95% confidence intervals were estimated. To assess the determinants of family functionality, the outcome variable (family functionality) was binary coded as ‘1’ for those who rated their families as dysfunctional (moderately and severely dysfunctional) and ‘0’ for those who rated their families as functional. The logistic regression model included only statistically significant variables (p<0.05). After at least 12 months of treatment, the treatment outcome variables consisted of patients with CD4 count decreased below baseline or above baseline and HIV viral load, categorised as undetectable, 20 – 1000 and >1000 copies/ml.

### Ethical consideration

Approval was obtained from the Committee on Human Research, Publication and Ethics (CHRPE), School of Medical Sciences (KNUST-SMS), Kwame Nkrumah University of Science and Technology and the Komfo Anokye Teaching Hospital, Kumasi, Ghana (CHRPE/AP/014/18). Participants voluntarily agreed to be recruited into the study and signed or thumb-printed an informed consent form. Their privacy and confidentiality were safeguarded throughout the study period and beyond.

## Results

### Demographic characteristics of study participants

A total of 304 study participants were recruited into the study. The mean age of participants was 41.8 (SD ± 9.9), with a minimum age of 22 years and maximum age of 78 years. Those who were females were 213 (70.1%). All the participants had a partner and were either married (n = 232, 76.3%) or cohabiting (n = 72, 23.7%). Participants with basic education were 198 (65.1%) and 237 (75.5%) in informal employment. The majority were Christians (n = 249, 81.9%). . (Table 1)

**Table 1:** Demographic characteristics of study partici-pants (N = 304)

Variable	Frequency n (%)
**Age (years)**	
**18 – 30**	37 (12.2)
**31 – 40**	112 (36.8)
**41 – 50**	95 (31.3)
**51 – 60**	46 (15.1)
**>60**	14 (4.6)
***Mean Age (years) (SD)***	*41.8 (±9.9)*
**Sex**	
**Male**	91 (29.9)
**Female**	213 (70.1)
**Relationship Status**	
**Married**	232 (76.3)
**Cohabiting**	72 (23.7)
**Level of Education**	
**Basic**	198 (65.1)
**Secondary**	47 (15.5)
**Tertiary**	24 (7.9)
**No Education**	35 (11.5)
**Religion**	
**Christianity**	249 (81.9)
**Islam**	53 (17.4)
**No Religion**	2 (0.7)
**Employment Status**	
**Informal Employment**	237 (78.0)
**Formal Employment**	38 (12.5)
**Unemployed**	29 (9.5)

### Clinical parameters and treatment outcomes

Out of the 304 participants, 96.4% (n = 293) tested positive for HIV 1 serotype (Table 2). At treatment initiation, 47.4% (n = 144) presented at WHO clinical stages I and II (early stages of infection) and 45.7% (n = 139) had CD4 count ≥ 200 cells/mm^3^ (non-AIDS stage). Participants' gender (p = 0.018), age (p = 0.002) and WHO Staging (p <0.001) were significantly associated with CD4 count at treatment initiation (Table 3).

**Table 2:** Clinical parameters and family functionality of study participants

Variable	Frequency n (%)
**HIV Type**	
**HIV 1**	293 (96.4)
**HIV 2**	1 (0.3)
**Both HIV 1 & 2**	10 (3.3)
**WHO Stage**	
**Stage I**	100 (32.9)
**Stage II**	44 (14.5)
**Stage III**	109 (35.9)
**Stage IV**	16 (5.3)
**Undetermined**	35 (11.5)
**Co-infection**	
**Hepatitis B**	14 (4.6)
**Hepatitis B & PTB**	2 (0.7)
**Pulmonary Tuberculosis** **(PTB)**	61 (20.1)
**Syphilis**	1 (0.3)
**None**	226 (74.3)
**CD4 at Treatment Initiation**	
**< 200**	110 (36.2)
**≥ 200**	139 (45.7)
**Not Done**	55 (18.1)
**CD4 at 12 months**	
**Above Baseline**	232 (76.3)
**Below Baseline**	32 (10.5)
**Not Done**	40 (13.2)
**Viral load at 12 months**	
**Undetectable**	218 (71.7)
**20 – 1000**	50 (16.4)
**> 1000**	36 (11.8)
**Family Functionality**	
**Functional**	214 (70.4)
**Moderately Dysfunctional**	41 (13.5)
**Severely Dysfunctional**	49 (16.1)

*PTB – Pulmonary Tuberculosis

**Table 3:** Relationship between demographic characteristics, clinical parameters and cd4 count

Variables	CD4 at Treatment Initiation		Chi-square/ Fischer's Exact P-Value	OR (95 % CI)	CD4 at 12 months after treatment		Chi-square /Fischer's Exact p-Value	OR (95 % CI)
	< 200 n (%)	≥ 200 n (%)			Above Baseline n (%)	Below Baseline n (%)		
**Gender**			5.6 (0.018)				0.023 a	
**Male (*ref*)**	44 (55.0)	36 (45.0)		1.00	76 (95.0)	4 (5.0)		1.00
**Female**	66 (39.1)	103 (60.9)		0.52 (0.31 – 0.90)	156 (84.8)	28 (15.2)		0.29(0.09 – 0.87)
**Age (years)** a			0.002				0.438	
**18 – 30 (*ref*)**	3 (13.6)	19 (86.4)		1.00	27 (79.4)	7 (20.6)		-
**31 – 40**	32 (36.4)	56 (63.6)		3.62 (0.99 – 13.18)	82 (87.2)	12 (12.8)		-
**41 – 50**	47 (56.0)	37 (44.0)		8.05 (2.21 – 29.27)	75 (89.3)	9 (10.7)		-
**51 – 60**	20 (48.8)	21 (51.2)		6.03 (1.54 – 23.57)	36 (90.0)	4 (10.0)		-
**>60**	8 (57.1)	6 (42.9)		8.44 (1.68 – 42.39)	12 (100.0)	0 (0.0)		-
**Relationship** **Status**			1.0 (0.310)				1.5 (0.220)	
**Married**	89 (45.9)	105 (54.1)	-	-	182 (89.2)	22 (10.8)		-
**Cohabiting**	21 (38.2)	34 (61.8)		-	50 (83.3)	10 (16.7)		-
**HIV Type** a			0.703				1.000	
**HIV 1**	106 (43.8)	136 (56.2)		-	223 (87.8)	31 (12.2)		-
**HIV 2**	0 (0.0)	0 (0.0)		-	1 (100.0)	0 (0.0)		-
**Both HIV 1 &** **2**	4 (30.8)	9 (69.2)		-	8 (88.9)	1 (11.1)		-
**WHO STAGE**			34.4 (<0.001)				0.067^a^	
**Stage I (*ref*)**	17 (20.2)	67 (79.8)		1.00	69 (82.1)	15 (17.9)	-	-
**Stage II**	20 (54.1)	17 (45.9)		4.64 (2.01 – 10.71)	38 (95.0)	2 (5.0)	-	-
**Stage III**	55 (62.5)	33 (37.5)		6.57 (3.31 – 13.03)	85 (91.4)	8 (8.6)	-	-
**Stage IV**	8 (61.5)	5 (38.5)		6.31 (1.83 – 21.74)	15 (100.0)	0 (0.0)	-	-

a analysed using Fischer's exact test

* Baseline = Treatment Initiation

The odds of a female HIV patient reporting to the health facility with CD4 count < 200 cells/mm^3^ (AIDS) at treatment initiation was lower (OR = 0.52; 95% CI = 0.31 – 0.90) when compared with their male counterparts (Table 3). Similarly, the odds of a patient reporting to the health facility at WHO stage IV (AIDS stage) with CD4 count < 200 cells/mm^3^ at treatment initiation was six times higher (OR = 6.31; 95% CI = 1.83 – 21.74) compared with patients who reported to the health facility at WHO stage I. The odds of a patient who is >60 years old recording a CD4 count < 200 cells/mm^3^ (AIDS) at treatment initiation was also higher (OR = 8.44; 95% CI = 1.68 – 42.39) when compared with patients who reported to the health facility between the ages of 18 – 30 years (Table 3).

After 12 months of treatment, 76.3% (n = 232) of the participants' CD4 count increased above baseline, suggestive of good immunologic response, while 10.5% (n = 32) of the participants` CD4 count decreased below baseline (suggestive of immunologic failure).^22^ This was significantly associated with the participant's gender (p = 0.023) (Table 3). The odds of a female HIV patient's CD4 count increasing above baseline after at least 12 months of treatment was lower (OR = 0.29; 95% CI = 0.09 - 0.87) when compared with male respondents (Table 3).

Approximately 71.7% (n = 218) of the participants attained the desired endpoint of undetectable viral load after 12 months of treatment. Those who recorded virologic failure (viral load > 1000 copies/ml) were 11.8% (n = 36) of study participants (Table 2). Participants' WHO stage was significantly associated with this outcome (p < 0.001) (Table 4).

**Table 4:** Relationship between demographic characteristics, clinical parameters and viral load

Variables	Viral Load at Treatment Initiation			Chi-square or Fischer's Exact P-Value	Viral load at 12 months after treatment			Chi-square or Fischer's Exact P-Value
	Undetectable n (%)	20 – 1000 n (%)	> 1000 n (%)		Undetectable n (%)	20 – 1000 n (%)	> 1000 n (%)	
**Gender**				0.185 a				2.2 (0.329)
**Male**	0 (0.0)	17 (22.1)	60 (77.9)		67 (73.6)	11 (12.1)	13 (14.3)	
**Female**	6 (3.2)	30 (16.1)	150 (80.7)		151 (70.9)	39 (18.3)	23 (10.8)	
**Age (years)** a				0.207				0.605
**18 – 30**	2 (6.1)	7 (21.2)	24 (72.7)		26 (70.3)	8 (21.6)	3 (8.1)	
**31 – 40**	3 (2.9)	23 (22.5)	76 (74.6)		84 (75.0)	19 (17.0)	9 (8.0)	
**41 – 50**	0 (0.0)	9 (11.4)	70 (88.6)		65 (68.4)	14 (14.7)	16 (17.2)
**51 – 60**	1 (2.7)	7 (18.9)	29 (78.4)		34 (73.9)	7 (15.2)	5 (10.9)	
**>60**	0 (0.0)	1 (8.3)	11 (91.7)		9 (64.3)	2 (14.3)	3 (21.4)	
**Relationship Status** a			0.371				0.160
**Married**	4 (2.0)	39 (19.7)	155 (78.3)		163 (70.3)	37 (15.9)	32 (13.8)	
**Cohabiting**	2 (3.1)	8 (12.3)	55 (84.6)		55 (76.4)	13 (18.1)	4 (5.5)	
**HIV Type** a				0.043				0.068
**HIV 1**	5 (2.0)	43 (17.1)	204 (80.9)		208 (71.0)	50 (17.1)	35 (11.9)	
**HIV 2**	0 (0.0)	1 (100.0)	0 (0.0)		0 (0.0)	0 (0.0)	1 (100.0)	
**Both HIV 1** **& 2**	1 (10.0)	3 (30.0)	6 (60.0)		10 (100.0)	0 (0.0)	0 (0.0)	
**WHO STAGE** a				0.015				<0.001
**Stage I**	4 (4.5)	12 (13.6)	72 (81.9)		85 (85.0)	8 (8.0)	7 (7.0)	
**Stage II**	1 (2.5)	6 (15.0)	33 (82.5)		28 (63.6)	6 (13.6)	10 (22.8)	
**Stage III**	1 (1.1)	19 (20.4)	73 (78.5)		67 (61.5)	30 (27.5)	12 (11.0)	
**Stage IV**	0 (0.0)	8 (57.1)	6 (42.9)		12 (75.0)	1 (6.3)	3 (18.7)	

*Excluded category; Not done (n =41)

a Analysed using Fischer's exact test

* WHO Stage; Undetermined (n=35)

*Baseline = Treatment Initiation

However, participants' gender (p = 0.329), age (p = 0.605), relationship status (p = 0.160), and HIV serotype (p = 0.068) had no significant association with CD4 count. The co-infection with the highest frequency was tuberculosis (n = 61, 20.1%), whilst approximately 74.3% (n = 226) of participants were not diagnosed with any co-infection (Table 2).

### Family functionality among study participants and relationship with treatment outcomes

Participants who rated their families as functional (Family APGAR score ≥ 7) were 214 (70.4%), and those who rated their families as severely dysfunctional (Family APGAR score < 3) were 49 (16.1%) (Table 2).

After at least 12 months of treatment, there was a statistically significant relationship between family functionality and viral load (p = 0.041) but no significant relationship between family functionality and CD4 count (p = 0.637) (Table 5).

**Table 5:** Relationship between treatment outcomes and family functionality

Variables	Family Functionality				Chi-square/Fischer's Exact P-Value

	Functional	Moderately Dysfunctional	Severely Dysfunctional	Total	
**Viral Load at 12 Months** **After Treatment**^a^					0.041
**Undetectable**	150 (68.8)	29 (13.3)	39 (17.9)	218 (100.0)	
**20 – 1000**	43 (86.0)	4 (8.0)	3 (6.0)	50 (100.0)	
**> 1000**	21 (58.3)	8 (22.2)	7 (19.5)	36 (100.0)	
**Total**	214 (70.4)	41 (13.5)	49 (16.1)	304 (100.0)	
**CD4 count at 12 months after** **Treatment**^a^					0.637*
**Above Baseline**	164 (70.7)	30 (12.9)	38 (16.4)	232 (100.0)	
**Below Baseline**	22 (68.8)	6 (18.8)	4 (12.4)	32 (100.0)	
**Total**	186 (70.5)	36 (13.6)	42 (15.9)	264 (100.0)	

*Excluded category; Not done (n =40)

a analysed using Fischer's exact test

Table 6 shows the results of the logistic regression analysis. In models 1 and 2, viral load 12 months after treatment was significantly associated with family functionality. The odds of participants whose viral load were between 20 – 1000 copies/ml at 12 months after treatment rating their families as functional was less (aOR = 0.32; 95%CI = 0.14 – 0.76) compared with participants who achieved undetectable levels of viral load at 12 months after treatment.

**Table 6:** Logistic regression model showing the relationship between viral load at 12 months after treatment and family functionality

Determinant	Model 1		Model 2	
	OR (95 % CI)	P – value	*aOR (95 % CI)	P – value
**Viral Load at 12 Months After Treatment**				
**Undetectable**	1.00		1.00	
**20 – 1000**	0.36 (0.15 – 0.84)	0.018	0.32 (0.14 – 0.76)	0.010
**> 1000**	1.58 (0.77 – 3.24)	0.217	1.86 (0.88 – 3.96)	0.105

* Adjusted for age and sex

## Discussion

The outcome of interventions for any illness largely depends on how early patients report and if appropriate therapy is initiated. Generally, HIV-positive patients report late to ART centres.^23^ In our study, female study participants reported to the treatment centres for initiation of therapy at an early stage of HIV infection. This buttresses the fact that there is gender disparity in patients who access ART as more women than men test and access treatment. This is attributable to women's more frequent contact with medical services and the increased efforts to intensify provider-initiated testing and counselling (PITC) for pregnant women.^23^ In Ghana, 66.9% of people who receive ARTs are women and 33.1% are men.^24^ This is comparable to our study in which 70% of study participants were women. In other parts of the world, HIV prevalence is higher in men than women. This is because the infection rate is common in heterosexual relationships and men who have sex with men (MSM), sex workers and their male clients.

In Europe (excluding Russia) the mode of HIV transmission includes but is not limited to the following; 46% through heterosexual sex, 26% from MSMs, 13% in drug users and less than 1% vertical transmission.^25^ A previous study reported that 50% of women who need ART receive treatment, but only 39% of men who need ART access it.^24^

One of the factors that promote early access to ART by women is active screening to prevent mother-to-child transmission of HIV.^24^,^26^

A patient's clinical stage at treatment initiation is related to the CD4 count. CD4 cells belong to a group of lymphocytes, also called CD4+ T cells, fighting infections within the human body.^27^ Its count may be influenced by sex,^28^,^29^ onset or existence of a co-infection, oral contraceptive pills, smoking habit and sleep patterns.^28^ A study in South Africa showed that females and younger patients had a better immune response to treatment.^30^In this study, an interesting observation was made in the variability of CD4 count.

At treatment initiation, factors significantly associated with CD4 counts were WHO clinical stage, age and sex. Even though females tended to have a higher CD4 count at treatment initiation than men, there was no significant relationship at six months after treatment. However, after 12 months of treatment, male participants were more likely to have CD4 counts above baseline. We surmise that this may be explained by decreased immunity because of changes in women's hormonal cycle during menstruation and pregnancy.

Since several factors influence CD4 count, viral load testing has overtaken its usefulness in clinical practice. Virologic response is, therefore, the major prognostic factor for ART outcomes.^4^,^6^

In this study, 12% of participants had a viral load > 1000 copies/ml after at least 12 months of treatment. Approximately 88.2% recorded viral ≤1000 copies/ml, which is higher than the 66% viral suppression in the general Ghanaian HIV population but below WHO's target to achieve 90% viral suppression among patients on ARTs.^31^,^32^ Worldwide, viral suppression after treatment ranges from 68% in Switzerland to 7% in China.^32^ The differences in the global reports may be attributed to the unavailability of ART centres in some countries, barriers to treatment retention, heterogenous data reporting, differences in healthcare systems and global socioeconomic dynamics.^32^,^33^

Apart from the WHO clinical stage, our participants' viral suppression had no significant association with their socio-demographic characteristics and other clinical parameters. A persistently high viral load may be due to treatment resistance, re-infection or drug-drug interactions; however, our study did not assess these.

We established that 70% of participants assessed their families as functional, which was associated with the viral load after 12 months of treatment. This highlights the importance of regular viral load testing in HIV care and that strong family support and good family function are essential in ensuring positive disease outcomes. This study considered participants' family functionality, an important but often neglected dimension of patient care. A functional family is associated with a high adherence to antiretroviral therapy, reduced incidence of loss to follow-up and improved treatment outcomes.^34^,^35^ A previous study has shown a significant correlation between family functionality and patients' health-related quality of life (HrQoL). The conclusion was that higher family function promotes better HrQoL.^36^ To our knowledge, there has been no published literature on Family APGAR and its association with HIV treatment outcomes.

This study was limited because data relied on participants' medical records including CD4 count, HIV clinical stage and viral load reports, some of which were unavailable. This was largely because of frequent stock out or laboratory equipment breakdown.

This provides useful feedback for the need to address health systems-related problems. The administration of Family APGAR was done once, after all the participants had been on treatment for a minimum of 12 months.

We recommend a future study that will assess patients' family functionality at the initiation of treatment and at the different stages of treatment to determine whether their APGAR scores vary with time and the associated factors.

## Conclusion

Our study participants' determinants of treatment outcomes were gender, WHO stage at treatment initiation and family functionality. The latter can be assessed using the Family APGAR, a simple tool, at the outpatient department and should be encouraged. When family dysfunction is detected, health care workers should try to reduce or eliminate it to mitigate any negative effects on ART outcomes. The ever-changing dynamics of HIV management call for a holistic approach which includes the entire family.
